# An integrated approach for spatial distribution of potentially toxic elements (Cu, Pb and Zn) in topsoil

**DOI:** 10.1038/s41598-021-86937-1

**Published:** 2021-04-08

**Authors:** Azadeh Vaziri, Ahad Nazarpour, Navid Ghanavati, Teimor Babainejad, Michael J. Watts

**Affiliations:** 1 Department of Soil Science, Khuzestan Science and Research Branch, Islamic Azad University, Ahvaz, Iran; 2 Department of Soil Sciences, Ahvaz Branch, Islamic Azad University, Ahvaz, Iran; 3 Department of Geology, Ahvaz Branch, Islamic Azad University, Ahvaz, Iran; 4 Inorganic Geochemistry, Centre for Environmental Geochemistry, British Geological Survey, Keyworth, UK

**Keywords:** Environmental sciences, Environmental impact

## Abstract

In this study, statistical analysis and spatial distribution were performed to compare raw data and centred log-ratio (*clr*) transformed data of three copper (Cu), lead (Pb), and zinc (Zn) potentially toxic elements (PTEs) concentration for 550 surface soil samples in Khuzestan plain. The results of both approaches showed that classical univariate analysis and compositional data analysis are essential to find the real structure of data and clarify its different aspects. Results also indicated that spatial distributions of raw data and *clr*-transformed data were completely different in three studied metals. Raw data necessarily shows the effects of anthropogenic activities and needs an additional evaluation of human health risk assessment for these three studied elements. Data obtained from *clr*-coefficient maps also demonstrated the role of geological processes in the distribution pattern of potentially toxic elements (PTEs). To improve the understanding of the implications for PTE pollution and consequences for human health, a RGB colour composite map was produce to identify the potential origin of PTEs from areas with higher than typical baseline concentrations.

## Introduction

There are two main sources of PTEs in soils: (i) natural background, which represents the PTEs concentration derived from parent rocks, and (ii) anthropogenic contamination, arising from the use of agrochemicals, organic amendments, animal manure, mineral fertiliser, sewage sludge disposal, atmospheric deposition and industrial wastes^1–4^. Soil contaminated with elevated PTEs concentrations is a serious environmental hazard due to their toxicity, persistence and potential for bioaccumulation in the urban environmnet, as well as providing a reservoir or sink for PTEs and other pollutants in urban areas^5,6^. Their elevated levels have negatively impacted human health via direct ingestion, inhalation and dermal contact absorption^7,8^. They can affect the central nervous system and may act as cofactors in other diseases. Different mechanisms, such as physical, chemical, and biological processes determine metal retention in soils^6,9,10^. Due to its high retention capacity, soil is often regarded as a sink for metals discharged into the environment^11–13^. It is important that detailed information on the distribution of PTEs in the environment, particularly in industrial towns, is available in an easily understandable format for policy decision makers so that soil contamination caused by industrial development can be assessed to inform practicable mitigation approaches, alongside public health monitoring^14,15^. In recent years, many studies have focused on the concentration, distribution and source identification of PTEs in industrial areas^16–18^. Based on spatial analysis, it was found that highly elevated metal concentrations were generally located in industrial and urban areas, along road networks and crowded commercial districts^19–21^. In contrast to PTEs in agricultural soils, those in urban soils have more possible sources, including vehicle emission, industrial discharge or waste incineration^22,23^. When considering toxicities severity, inability to regeneration, high fetal mortality, mutation, offspring abnormalities are the most important consequence of PTEs in animals^24–26^.

To better understand the extent of soil contamination from PTEs to inform appropriate management/remediation, it is necessary to have utilise spatial characteristics to employ geostatistics or mapping systems as usable tools^27,28^. A geostatistical approach can help to identify contamination sources and the spatial distribution of PTEs in the environment^29–31^. These techniques are ideal for the evaluation of interactions between PTEs released to the environment and recipient environment based on the spatial information of pollution distribution sources, processes affecting pollutants distribution and population density^32–34^. The output of geostatistics techniques provided the scientific basis for better evaluation and management of the environment^8,18,25^. Since the 1980’s, geochemical data for geostatistical consideration have been based on log-transformation calculations^35–37^. Based on recent findings, the study of the concentration of the controlling characteristics of geochemical data and their distribution in the transformed state could result in reaching differing interpretations^38^. However, geochemical data should be considered as a sample of composition and closed data, which should be opened before data processing^39,40^.

To date, studies on PTE concentrations in soil from the Khuzestan plain of Iran have included a limited pollution level assessment by classic statistical procedures and statistical methods such as the cumulative probability and box plot and map analysis based on log-transformation^41–43^.

Therefore, the aim of this study was to statistically evaluate and determine the spatial distribution pattern of three PTEs (Pb, Zn, and Cu) in surface soil from the Khuzestan plain by univariate data and compositional data analysis. These elements were recognised as the principle PTE with a high potential risk for human health in the Khuzestan plain^44^. In order to evaluate the potential effect of these elements on this environment an RGB map was prepared to provide a spatial representation of contamination for Pb, Zn, and Cu soil concentrations in the Khuzestan plain, which will provide a usable tool for policy decision makers.

## Materials and methods

**Figure 1:**
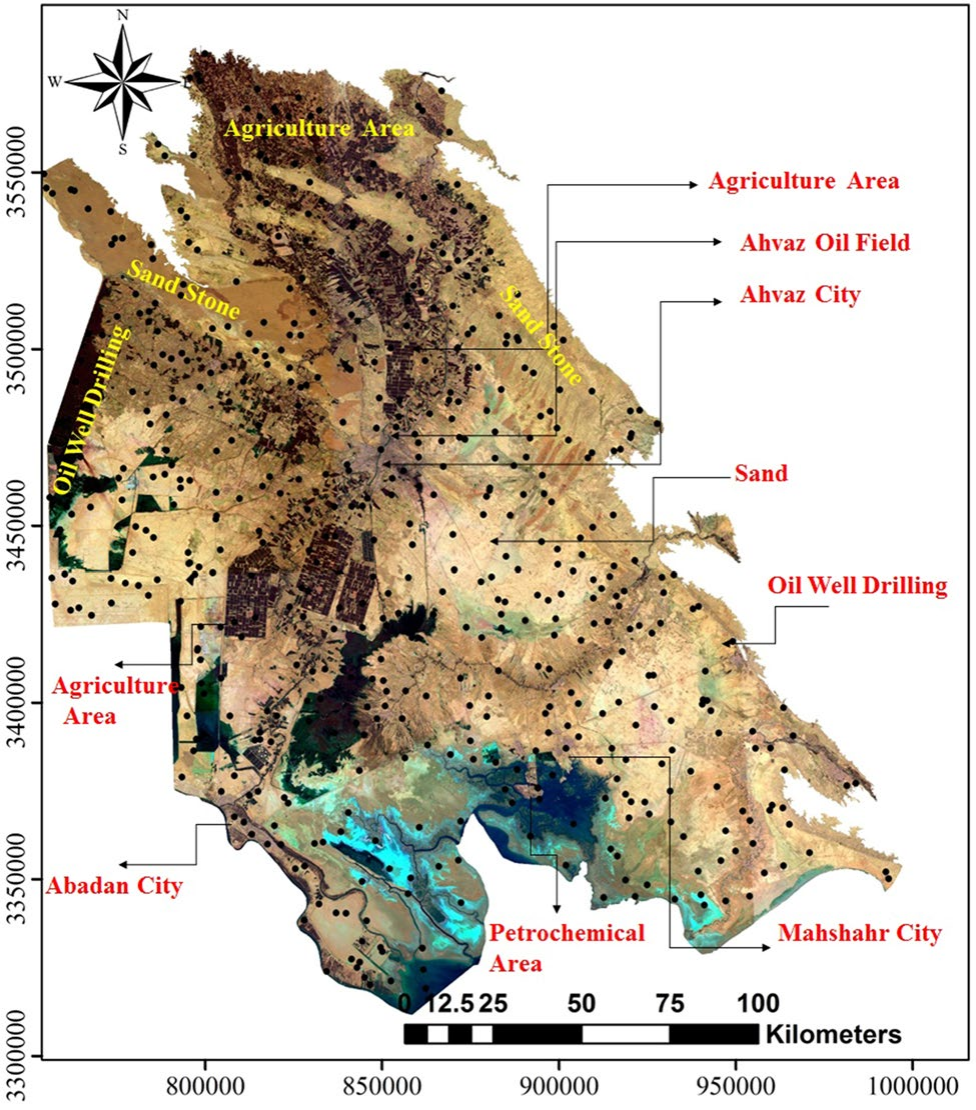
Satellite image of study area (Khuzestan plain) and sampling points, the image was made by ArcGIS10.2, background from Google Earth (Image: Google, Landsat/Copernicus).

The study area is in the southwest of Iran in Khuzestan province, covering an area of about 63,213 km^2^ and located between longitudes 48° and 49.5° E and latitudes 31° and 32° N with almost 4,000,000 inhabitants (Fig. 1). Elevation ranges from 0 to 3737 m, with a cold (in the north) and tropical (in the south) climate, with mean maximum summer temperatures (July) about 50 ºC. The climate of the study area is considered to be arid and humid. This area is part of the Zagros orogenic belt. This belt is the product of three major geotectonic events during subduction between the Arabian and Iranian plates^45^. The belt consists of their parallel tectonic zones from NE to SW: (1) the volcanic–plutonic zone (Urumieh– Dokhar belt); (2) the Sanandaj-Sirjan metamorphic zone; (3) the Zagros fold belt^45^. Sedimentary rocks consist of chemical-biochemical limestone to clastic sandstone—Conglomerate ages ranging from Cretaceous to quaternary are occupied Khuzestan province. Rapid erosion in the Zagros area is combined with high water flow resulting in a large sediment load. The rock fragments and minerals derived from erosion of the banks of the rivers and its tributaries are transported south and accumulate after the break in slope where the rivers reach the flat plain (e.g., Ahvaz city). The surficial distribution of sediments shows miscellaneous layers and mixture of sands, silts and hard muds. Generally, Khuzestan plain is characterised by the predominance of alluvial and sedimentary rocks of both chemical and detrital origins. The sand and much of the coarse silt alluvial are typically composed of quartz. The fine silt and clay fractions are dominated by clay minerals. The sediment minerals were subjected to sorting by size and specific gravity, as well as some chemical dissolution during transportation.

### Soil sampling and laboratory analysis

**Figure 2:**
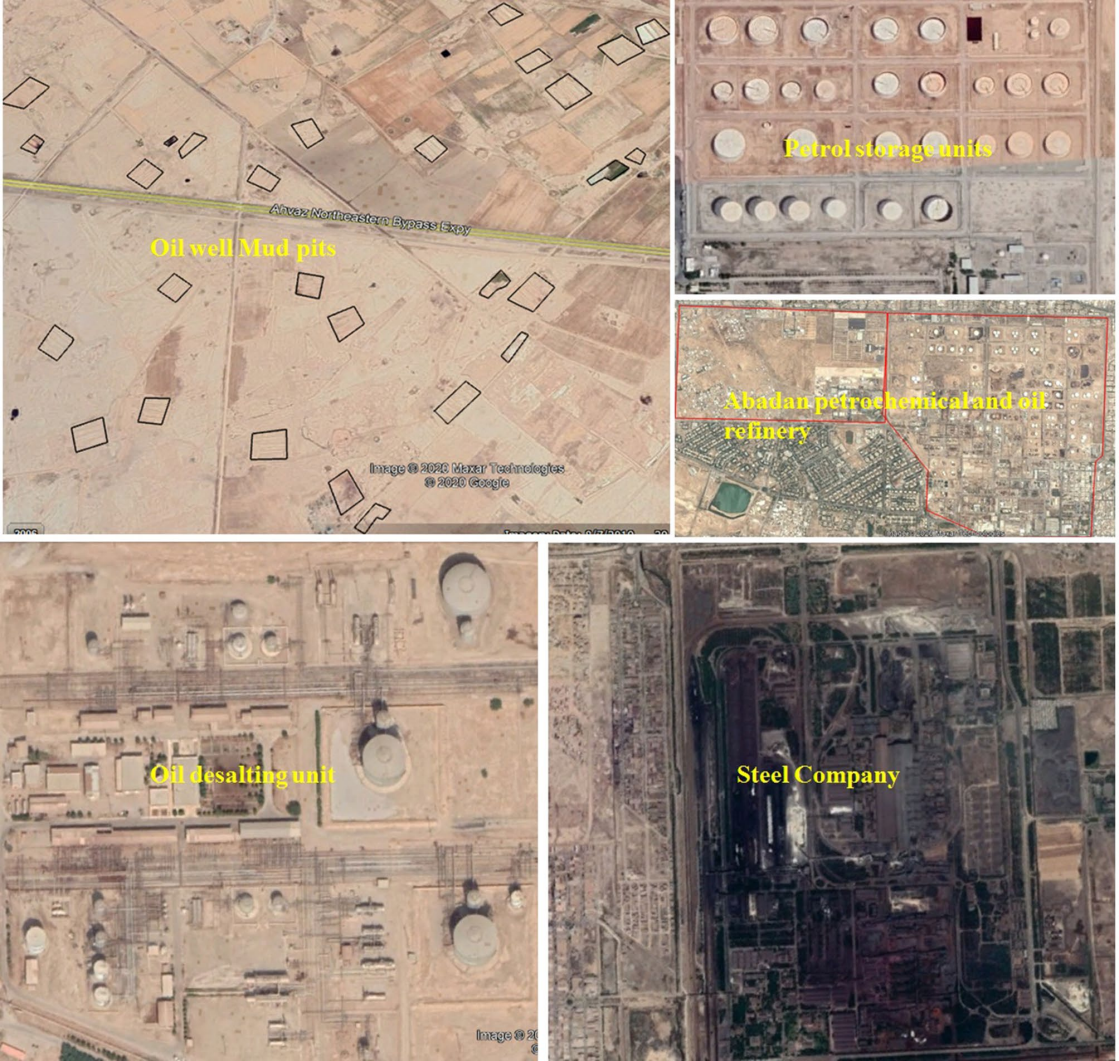
Satellite image of some oil related steel industries in Khuzestan plain, the image was made by ArcGIS10.2, background from Google Earth (Image: Google, Landsat/Copernicus).

In this study, 550 soil samples were collected from the Khuzestan plain in 2016, which included urban, suburban, agricultural and oil field and industrial zones (Fig. 2). Approximately 1.5 kg of soil was gathered from each sampling point from 5 to 15 cm depth after sieving to < 200 mm and removal of superficial plant material based on the international instruction of GOREGS Geochemistry Group^46^. Each sample was a composite of three collected samples in 5 m interval. Samples placed in a 60° C oven for 24 h to obtain 0.15 mm particles. Then in order to extract metal from samples, 1 g of each dried sample was heated in a rubber balloon containing 4 ml nitric acid (with 1 + 1 weight ratio) and 10 ml Hydrochloric acid (with 1 + 4 weight ratio) in 95° C for 2 h to a final matric of 65% HNO_3_/ 40% HCl, for subsequent PTE analysis by ICP-OES (Spectro Arcos, Germany). We measured the control and duplicate the samples with a precision of 4 to 6%, and reference materials NIST 2710 with an accuracy of 100 ± 8% (n = 30), for Quality Assurance (QA) and Quality Control (QC). The duplicate soil samples' precision was 5 to 7% and less than 5% (Table 1).

**Table 1:** Relative percentage difference and accuracy of analytical results of studied PTEs.

	RPD (%)	Accuracy (%)
Pb	2.83	3.69
Cu	5.35	4.37
Zn	6.32	3.86

### Compositional nature of geochemical data

The compositional nature of geochemical data considered an important issue and should be considered before any geostatistical analysis of geochemical data^47–50^. Composition or closed data, is a series of data in which the variants are not independent and represented as percent or ppm or a part of the total^40,51^. In the classical definition, each data raw named as observation in which the total analysed the variation of observation is a constant number (such as, 1, 100 or 106). Although regarding the major property of this data as scale instability and its sub-unit integration necessarily does not need to establish a fixed total condition^52^.

Compositional data have properties that make it difficult to apply a standard statistical method. The Euclidean space is not suitable for compositional data and limitation, whilst the constant sum of these data indicated a certain geometry which so-called Aitchison geometry in a simple environment^53^. In order to use standard statistical methods, these data should be transformed in a suitable way such as relativistic logarithm transformations presented by^54^. The space of sample data or simplex for a partial D combination X = (X_1_,…., X_D_) or subsequent –D defined as Eq. (1):

1 $${S}^{\mathrm{D}}=\left\{X=\left({x}_{1},\dots ,{x}_{D}\right)|{x}_{i}>0,i=\mathrm{1,2},\dots , D; \sum_{i=1}^{D}xi=K\right\}.$$

Using three log-ratio transformation additive log-ratio (*alr*)^55^, centered log-ratio (*clr*)^43^, and isometric log-ratio^56^, (*ilr*) these data could be transformed to the Aitchison space. For instance, *clr* transformation led to a multivariate observation in D-1-dimension space, and defined according to following Eqs. (2) and (3):

2 $$ilr\left(x\right)=\left({\mathrm{z}}_{1},\dots , {\mathrm{z}}_{\mathrm{D}-1}\right)=$$

3 $$\sqrt{\frac{\sqrt{D-i}}{D-i+1 }} \mathit{ln}\frac{{x}_{i}}{\sqrt[D-i]{\prod_{j=i+1}^{D}{x}_{j}}} ,\mathrm{ for }i=1,\dots ,D-1.$$

Data interval in this geometry is the Aitchison space, calculated according to Eq. (4) for both $X=\left({x}_{1},\dots , {x}_{D}\right)$ and $Y=\left({y}_{1},\dots ,{y}_{D}\right)$ compositions:

4 $${ d}_{A}\left(X,Y\right)=\sqrt{\frac{1}{D}\sum_{\mathrm{i}=1}^{D-1}\sum_{\mathrm{j}=\mathrm{i}+1}^{\mathrm{D}}(ln\frac{{\mathrm{x}}_{\mathrm{i}}}{{\mathrm{x}}_{\mathrm{j}}}-ln\frac{{y}_{i}}{{y}_{j}}{)}^{2}}.$$

Isometric property of *clr* conversion mean for two X and Y compositions, Eq. (5) is established between both the Aitchison and Euclidean space.

5 $$ d_{A} \left( {X,Y} \right) = d_{E} \left( {clr\left( X \right),clr\left( Y \right)} \right){ } .$$

To correct the interpretation of diagrams using univariate scalars (e.g. histogram, boxplot governed by the Euclidean relationship), there is another relation proposed by Filzmoser, et al.^57^) to the univariate conversion of data^53^ using the following relation for each xi based on Eq. (6):

6 $$ Z_{i} = \frac{{\sqrt {{\text{D}} - 1} }}{{\text{D}}}ln\frac{{x_{i} }}{{\sqrt[{D - 1}]{{\mathop \prod \nolimits_{j}^{D} = 2^{{x_{j} }} }}}}{ } .$$

Average in these types of data calculated according to Eq. (7):

7 $$ {\overline{\text{X}}} = clr^{ - 1} \left( {\frac{1}{n}\mathop \sum \limits_{i = 1}^{n} clr\left( {x_{i} } \right)} \right){ } .$$

There is a different definition for compositional data variance which metric variance or total variance or global variance are between them and the average of distance squared from the data center in company with corrected degrees of freedom is the variance of general data and obtained by Eq. (8):

8 $$ var\left( X \right) = \frac{1}{{{\text{n}} - 1}}\mathop \sum \limits_{{{\text{i}} = 1}}^{n} {\text{d}}_{{\text{A}}}^{2} \left( {x_{i} ,{ }\overline{X}} \right){ }. $$

Therefore, standardising combined data is different from typical statistic methods. Firstly, combination data have a non-dimensional common scale and thereby standardising with the usual process caused loss of important data which included variability in data. Secondly, normal averaging produced negative values that the interpretation of the combination average power of reverse combined variance square according to Eq. (9):

9 $$ Z = { }\frac{1}{{\sqrt {var\left( X \right)} }} \odot \left( {{\text{X}}\Theta {\overline{\text{X}}}} \right){ } $$

where ${ } \odot$ in this relation is the power and $\Theta$ is the reverse function in simplex space^58^.

### Data analysis

In this study for data analysis, both log-transformed data and compositional data statistically and spatially were analysed^53,59^. The *clr*-transformation method was used to analyse combination data^41^. Also a free open source CoDaPack software was used to transfer raw data to *clr*-transformation data.

The statistical summary of raw data analysis results and *clr*-transferred data including minimum, maximum, percentile (10, 25, 50, 75, 90, 95, 98), mean, standard deviation, median absolute deviation (MAD), which are useful to represent variability and central tendency of data structure are indicated in (Tables 2 and 3). For *clr*-transformed variables as variance increased, the effect of these variables raised on multivariate data^38,40,53^.

**Table 2:** Summary of the main statistical parameters (unit of measure (UM), minimum (Min.), maximum (Max.), percentiles (P) 10, 25, 50, 75, 90, 95, 98, mean, standard deviation (SD), interquartile range (IQR) and median absolute deviation (MAD) of Pb, Zn and Cu measured contents.

	Min	P10	P25	P50	Mean	P75	P90	P95	P98	Max	SD	IQR	MAD
Pb	10	18	33	45	64.38	82	94.1	99.6	298.36	610	64.59	49.75	22.36
Zn	25	40	64	98	102.55	135	163	12	1.22	493	53.39	71	39.21
Cu	8	12	23	39.5	50.95	56.75	67	69	334.22	865	76.65	32.75	36.76

**Table 3:** Summary table of the main statistical parameters (minimum (Min.), maximum (Max.), percentiles (P) 10, 25, 50, 75, 90, 95, 98, interquartile range (IQR) and median absolute deviation (MAD)) of clr-transformed values of Pb, Zn, Cu.

	Min	P10	P25	P50	P75	P90	P95	P98	Max	IQR	MAD
Pb	− 1.5	− 0.06	0.19	0.54	0.8	1	1.22	1.28	1.32	0.75	0.41
Zn	− 1.7	− 0.73	− 0.38	− 0.05	0.23	0.46	0.63	0.775	1.7	0.61	0.18
Cu	− 1.74	− 1.58	− 0.75	− 0.35	0.07	0.15	0.31	0.98	1.24	0.66	0.29

Means and standard deviation were not presented for *clr*-transformed data because of incompatibility to compositional geometry as a criterion for central tendency and dispersion and its behaviour is based on Euclidean geometry, while compositional data do not relate to classical Euclidean space^38,40,59^.

**Figure 3:**
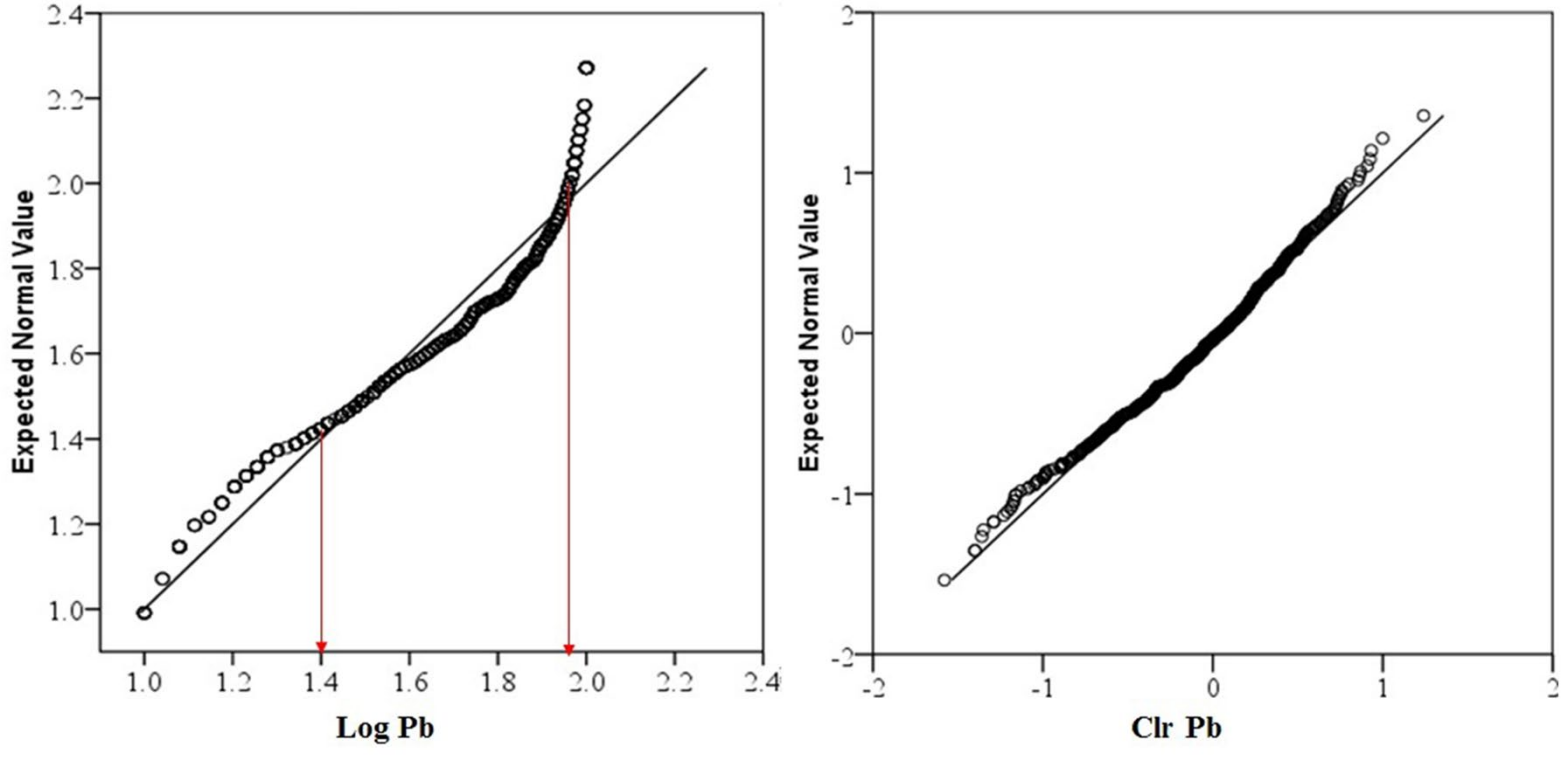
Q–Q plot of: **(a)** Pb original log-transformed data; **(b)** Pb clr-transformed data.

**Figure 4:**
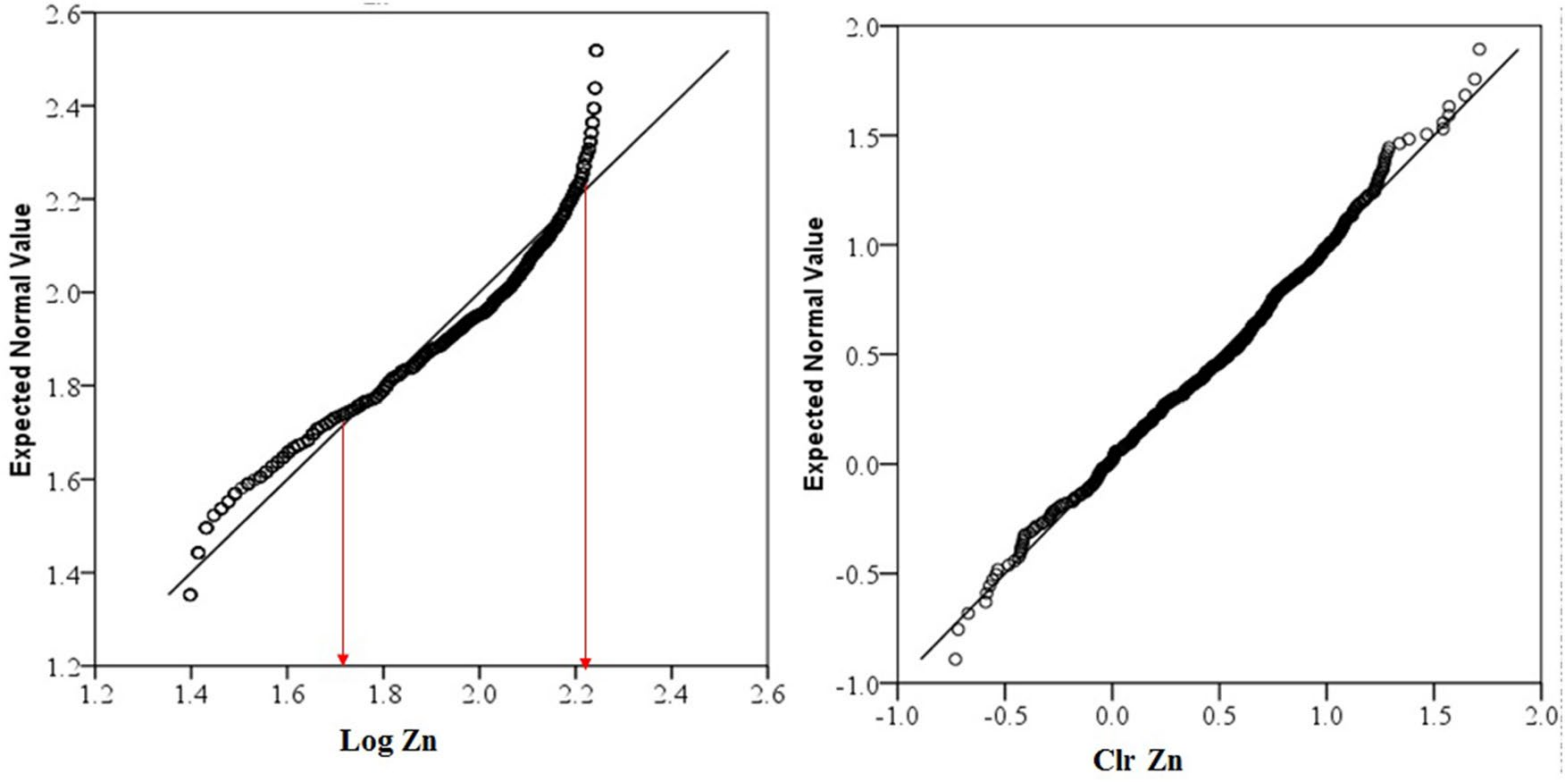
Q–Q PLOT of: **(a)** Zn original log-transformed data; **(b)** Zn clr-transformed data.

**Figure 5:**
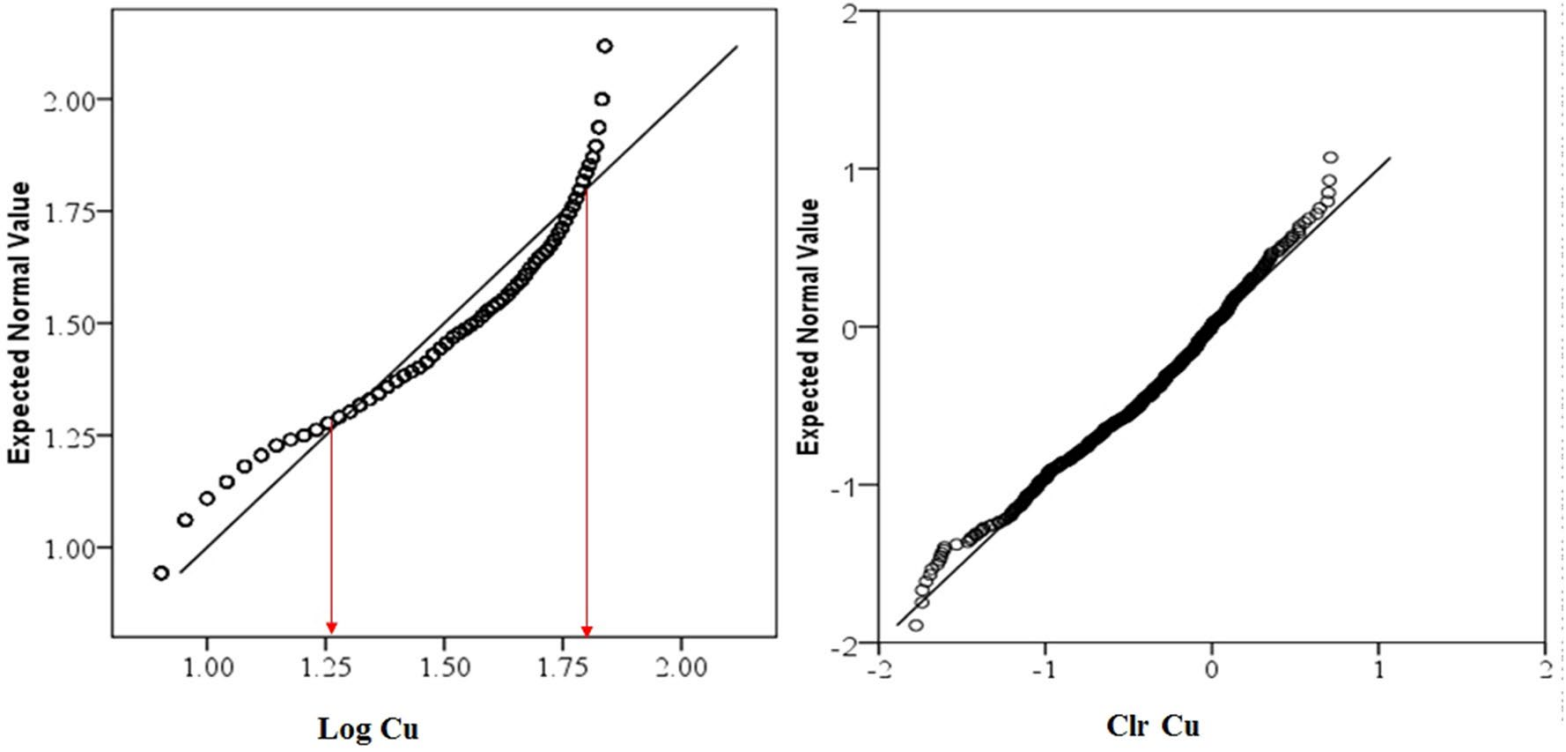
Q–Q PLOT of: **(a)** Cu original log-transformed data; **(b)** Cu clr-transformed data.

**Figure 6:**
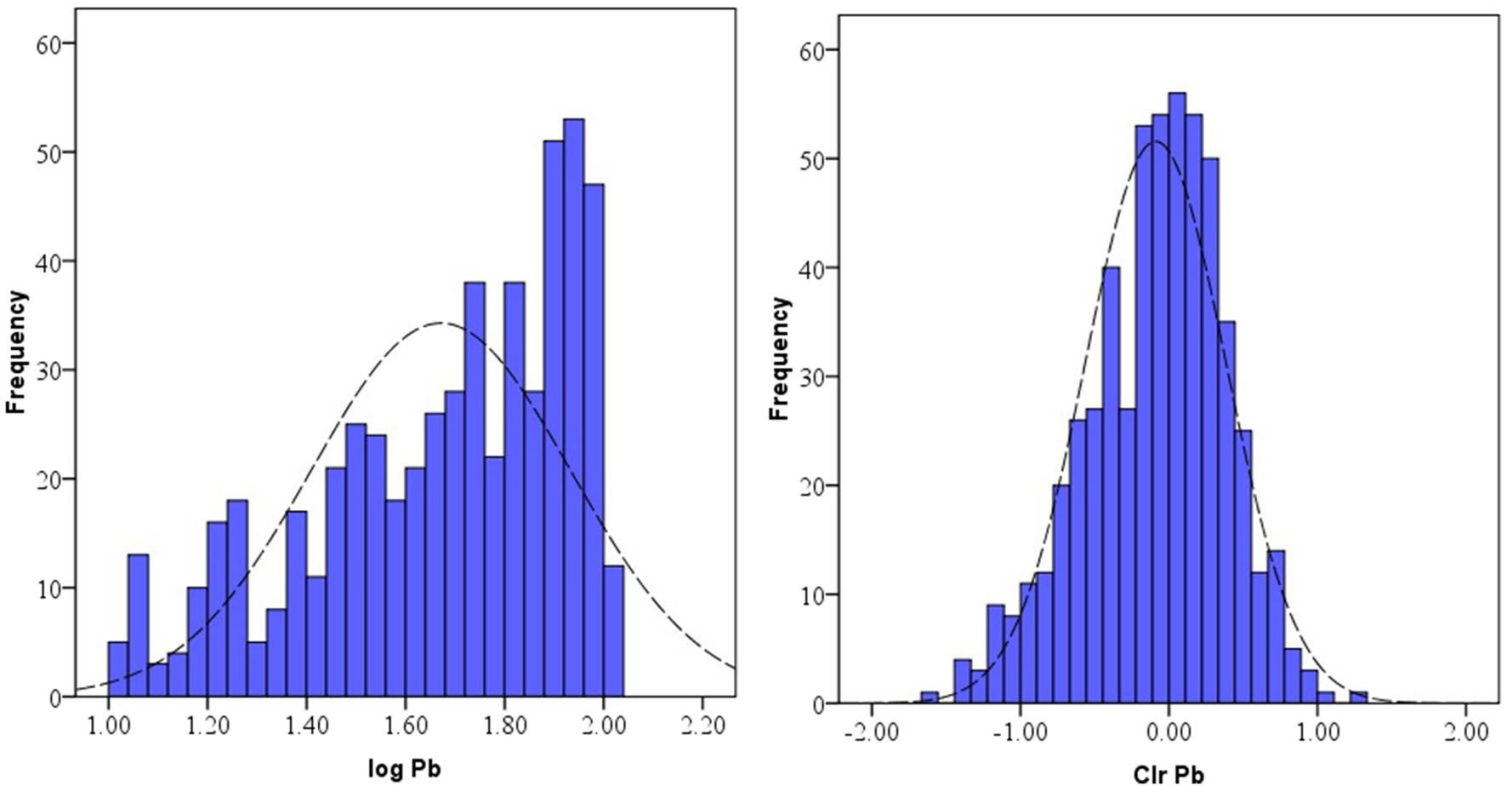
EDA plots for: **(a)** Pb original log-transformed data; **(b)** Pb clr-transformed data.

**Figure 7:**
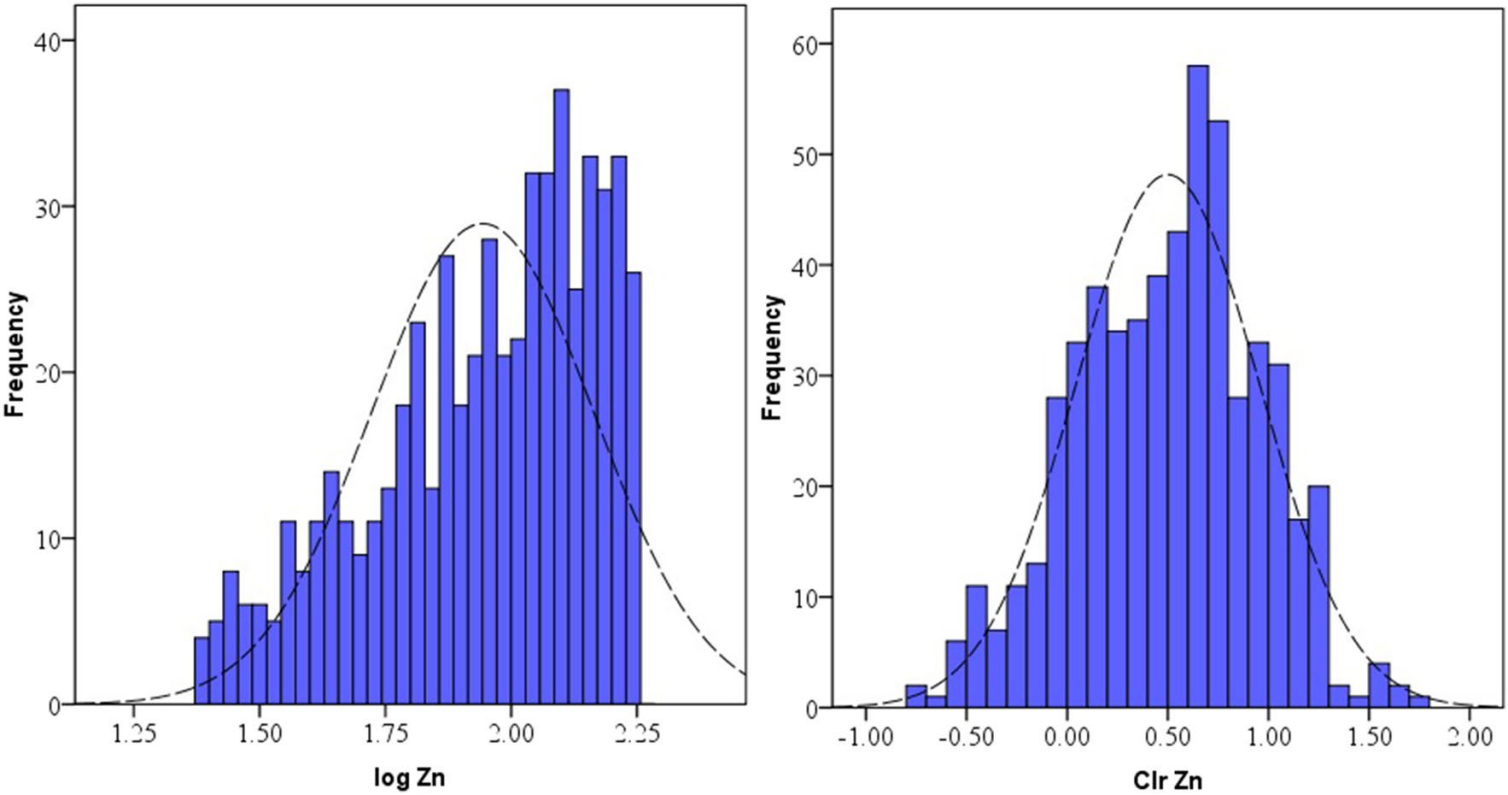
EDA plots for: **(a)** Zn original log-transformed data; **(b)** Zn clr-transformed data.

**Figure 8:**
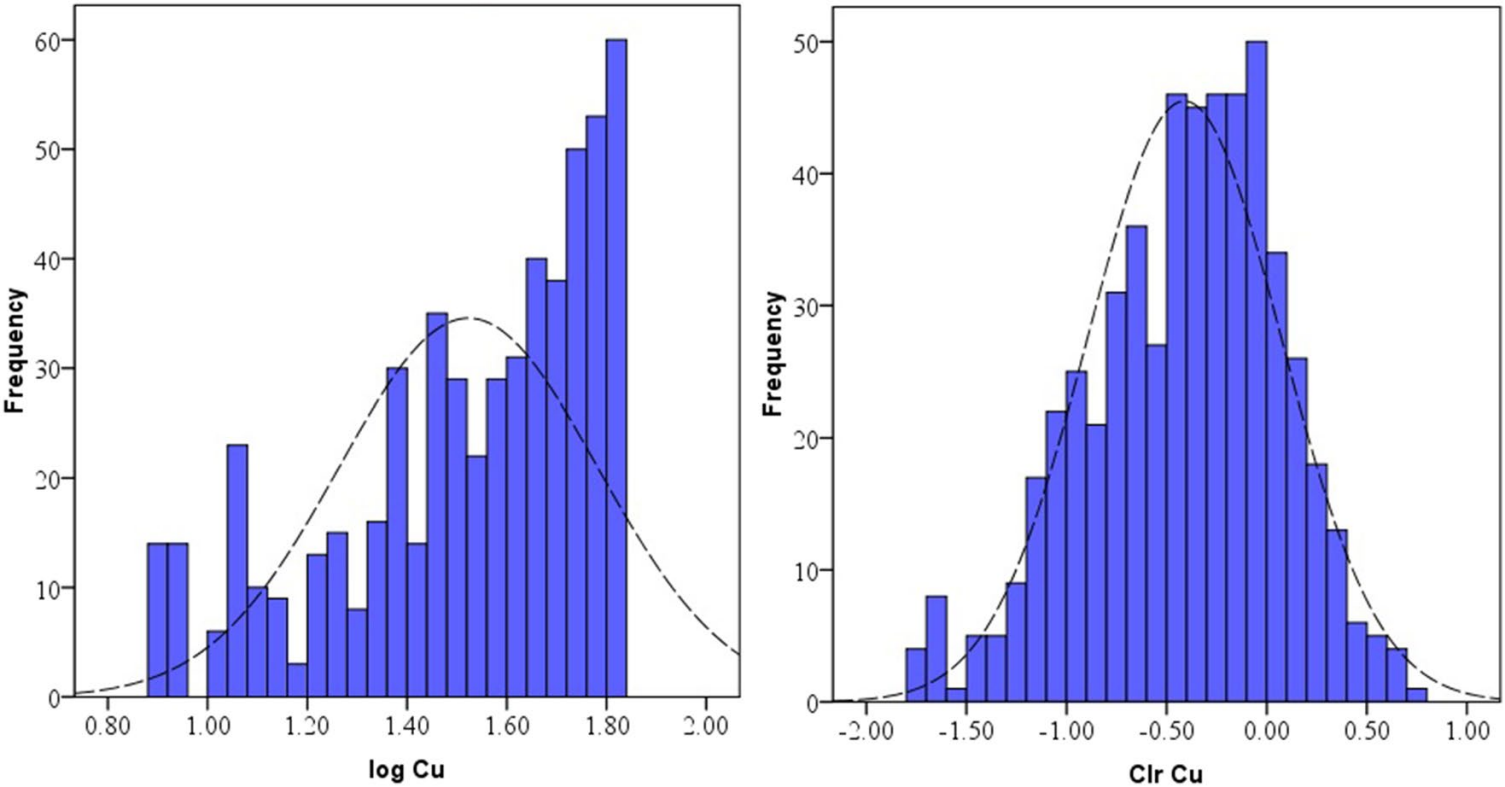
EDA plots for: **(a)** Cu original log-transformed data; **(b)** Cu clr-transformed data.

Data distribution and normality status of studied PTEs of element concentrations were presented by a detail in cumulative Q–Q plot of raw and *clr*-transformed data (Figs. 3, 4, 5a,b) and exploratory data analysis (EDA) plot of log-transformed and clr-transformed of studied PTEs are presented in Figs. 6, 7, 8a,b. Vertical red lines in Q–Q plots of log-transformed data (Figs. 3a, 4a and 5a) indicate threshold values established by the Iranian soil quality guideline for assessing human health risk and local background of study area^60^. Each EDA plot is a combination of histogram, one- dimensional scatterplot and it is considered one of the best graphical displays of data distribution^38^.

**Figure 9:**
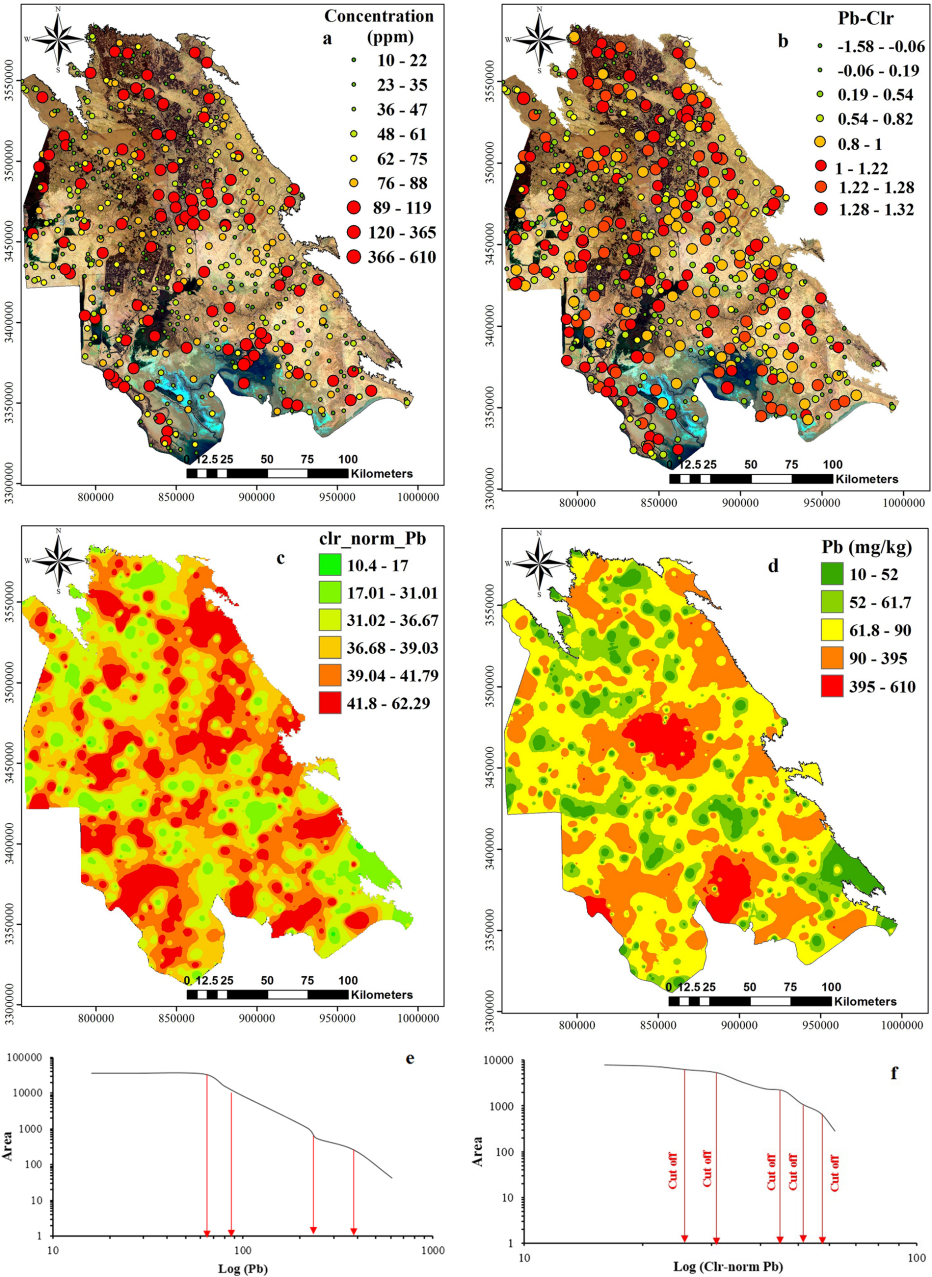
Interpolated (MIDW) maps for **(a)** Pb raw data; **(b)** Pb clr-transformed data, and corresponding plots of C-A model for map classification. the image was made by ArcGIS10.2, background from Google Earth (Image: Google, Landsat/Copernicus).

**Figure 10:**
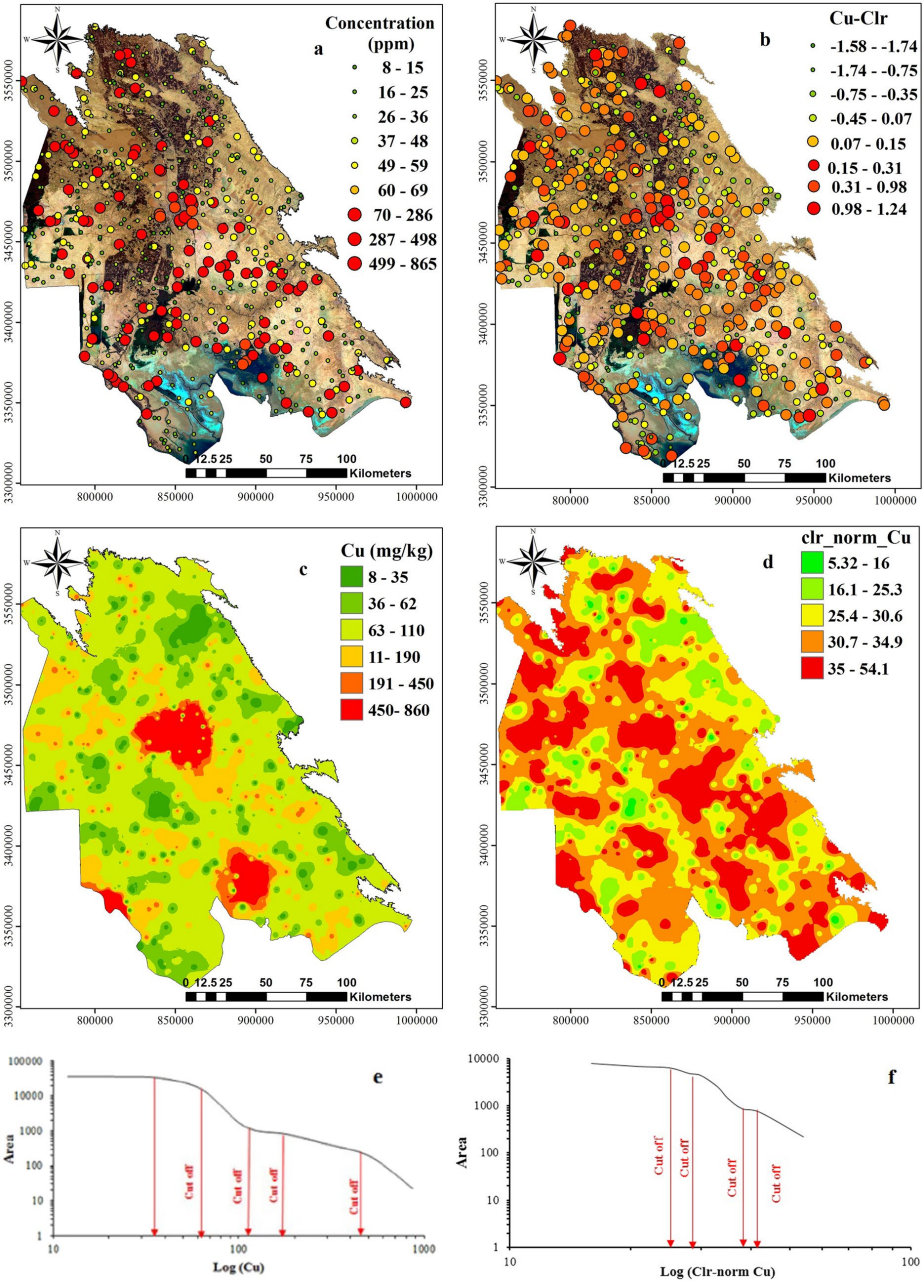
Interpolated (MIDW) maps for **(a)** Cu raw data; **(b)** Cu-Clr-transformed data, and corresponding plots of C-A model for map classification, the image was made by ArcGIS10.2, background from Google Earth (Image: Google, Landsat/Copernicus).

**Figure 11:**
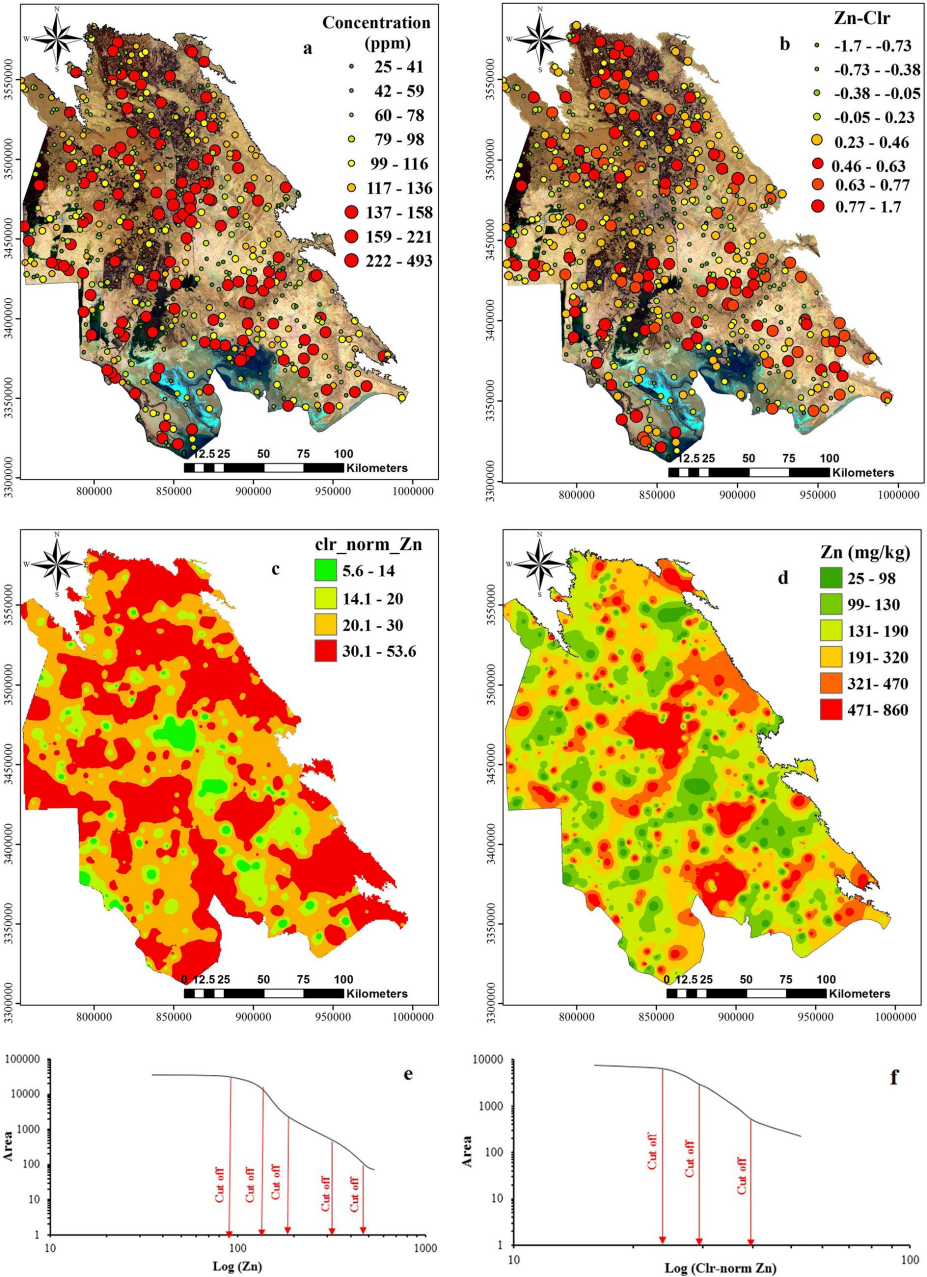
Interpolated (MIDW) maps for **(a)** Zn raw data; **(b)** Zn-Clr-transformed data, and corresponding plots of C-A model for map classification, the image was made by ArcGIS10.2, background from Google Earth (Image: Google, Landsat/Copernicus).

Dot maps (Figs. 9, 10, 11a,b) and interpolated maps (Figs. 9, 10, 11c,d) were produced to visualise the spatial data structure. Dot maps were reclassified based on percentiles calculated in Tables 2 and 3 by ArcGIS software^59,61,62^. Calculated concentrations were interpolated by GeoDdas software and obtained using multifractal inverse distance weighted (MIDW) method (Figs. 9, 10, 11 e,f).

Concentration-area fractal method is one of the conventional methods to display distribution of element concentration in an area and depicting iso-concentration contour maps in the studied region. If each contour value considered ${\uprho }$, a power equation could be represented as the following relation^59,63–65^. D is the fractal dimension corresponded to different domains of ${\uprho }$.

10 $$ A\left( { > \rho } \right) \propto \rho^{D} .$$

Depicting area changes against concentration in Log plot, the dimension of each geochemical population could be calculated through the straight-line gradient. Obtained fractal dimension demonstrated coverage area of available data, as fractal dimension is the last fitted line with high concentration and typically have the lowest value indicating a lower area of high concentration samples.

In this research pixel-based method was used for C-A model in order to separate geochemical populations. In this way, raw geochemical samples were firstly prepared in ArcGIS with raster maps with cell size of 500 × 500 m, then single element geochemical surface maps were created. By applying the classification method in an attribute table, which are common functions in GIS, the available pixel number was determined at each level. By applying the C-A fractal method on current pixel values, threshold estimation performed with more accuracy.

Before MIDW interpolation, because *clr*-transformed data contains negative values and negative values cannot represent accurate conception from the data situation in the concentration-area method, therefore, before running this model data should be normalised (1–100) (Figs. 9, 10, 11e,f). The Min–Max normalisation method was selected because this factor is a linear conversion which preserves the data structure^40,66,67^. MIDW Interpolated maps were classified by estimation confines obtained by the C-A fractal method^59,68^. With the help of log plot of C-A and threshold methods, we could divide interpolated maps based on different pixels’ population and showed them by different colors on the map.

Colours on maps provide a visual representation of different units with various geochemical properties (e.g. mineralisation events, surface geochemical element concentration, surface weathering), as represented by^63,69^. In interpolated maps, *clr*-transformation and C-A classification performed by normalised values (1–100). Then interpolated maps of Pb, Zn and Cu were compared to the contamination threshold (CSC) of standard soil in Iran. Each raster map with the corresponding element threshold value was divided for residential use: 100 mg/kg for Pb, 120 mg/kg for Cu and 150 mg/kg for Zn.

In this approach, three pollution maps were obtained for which each pixel above 1 represents hazardous conditions for human health. To prepare an overlay map showing elevated concentrations of the three studied elements, a colour composite image processing method was required (Fig. 5). This method is based on the RGB (Red, Green, Blue) colour model and was widely used to process satellite images and geochemical mapping showing three differing distribution patterns^40,70,71^. To process a digital image, every primary colour had an integer number from 0 to 225 and saved based on colour intensity. Each colour of the pixel in the satellite image is the result of values (Red, Green, Blue) which indicated colour intensity to produce an RGB combined colour. In this study, with the help of a colour composite tool box in ArcGIS software, Zn, Cu and Pb maps were considered as monochromatic maps. Three types of pure colours (Red, Green and Blue) were added to generate a special colour composite map where each pixel value is indicative of three colours. For improved resolution of colour distribution in the composite colour map, a monochromatic map of Zn, Pb and Cu was normalised between 0 and 1 values. In this approach values above 1 were classified in the RGB space between 0 to 225 in three maps.

## Results

The main minerals available in studied area soil included clay, quartz, and carbonate, and subsidiary minerals are mainly feldspar and gypsum. The order of the mean frequency of minerals in the soil included clay < 39.55, quartz < (19.32%), carbonate < (16.91%), gypsum < (11.32) Alkali Feldspar < (9.14%). The percent of calcite, illite, and Smectite is demonstrative of weak soil chemical weathering and collected calcium carbonate.

Lead concentrations in analysed soil samples ranged from 10 to 610 mg/kg with a mean of 64 mg/kg, significantly greater than values reported for European soils (5.3–970, median: 15 mg/kg^72^; Italy (2.55 to 204 mg/kg, mean: 32 mg/kg^73^; Netherland (36 mg//kg mean—VROM, 2000—; China (with 26 mg/kg averagely) and Worldwide soils (14 mg/kg^74^).

The Cu concentration in surface soil samples varied from 8.5 to 865 mg/kg with mean value (75.95 mg/kg), which this range of concentration is higher than measured values in Europe soil (0.81 mg/kg to 256 with the means of 62.2 mg/kg)^72^ and Italy surface soil (3–215 mg/kg, mean: 22 mg/kg)^64^, world soil with mean value of 62 mg/kg^74^, Netherlands soil means (36 mg/kg)^75^ and background soil value of China (26 mg/kg average).

The Zn concentration ranged from 25 to 493 mg/kg, with mean value (102.5 mg/kg). Zn concentration in studied area was higher than reported values for Europe (37 to 396 mg/kg, mean value of 81 mg/kg)^72^, Italy surface soil (with 3–2900, mean: 48 mg/kg)^73^, world soil (25 mg/kg)^74^, Netherland soil mean (85 mg/kg)^75^ and background value of Chinese soil ( 74.2 mg/kg average).

Table 3 provides the descriptive analysis/classification of studied PTEs in the sub-compositional state. High concentration metals rather than the geometric mean had a positive sub-composition with *clr*-value^76^. For Pb with the mean value of the *clr*-value (*clr* mean) of 0.54, while 25-percentile of Pb with the positive value indicated that more than 75% of Pb values were above the geometric mean of three studied PTEs elements. The *Clr* median value of Zn and Cu showed that more than 50% of these values were lower than the geometric mean of sub-composition data.

The lowest amount for sub-composition variability was for Zn and showed the lowest median absolute deviation (MAD) from the mean (0.18), while Pb and Cu exhibited the same variability of 0.41 and 0.29 absolute deviation from the mean, respectively. The IQR and MAD values showed that the data variability effect of Cu and Zn was moderately higher than Pb. *Clr* cumulative distribution graphs of Pb and Zn (Figs. 3, 4, and 5b) showed obvious changes in the distribution of slope breaks and demonstrated different geochemical populations and geological processes in which higher values were anomalous.

## Mapping

Concentrations higher than > 89 mg/kg of Pb in 10% of samples mostly belonged to areas with anthropogenic activities, such as petroleum, including oil well drilling, oil desalting units, oil storage units, drilling mud pits (mostly oil based mud), petrochemical and oil refinery and steel industries, along with intensive traffic (Fig. 11a). The interpolated map showed that the lower concentration of Pb (< 18 mg/kg) is mostly in the Khuzestan plain, especially in arid areas and far from large industries and covered largely by windblown sand. Rock outcrops were also found in conglomerate and sandstone. Median concentration zones for Pb (19 to 45 mg/kg) were without detectable trends, comparable to agricultural and sugarcane industries. On checking the study area, the lack of large industries such as oil associated activities agricultural activity is the principle activity in raising PTEs concentration in this area. The most widely used fertilisers in Khuzestan plain are urea, ammonium phosphate and Triple Super-phosphate. The role of agriculture as the main factor for elevated PTEs in soil was also reported by other studies (Li et al., 2008; Bai et al., 2010). The Pb concentrations between 46 and 90 mg/kg in the main urban areas were located in Ahvaz, Mahshahr, Abadan and Bandar-e-Emam, while concentrations higher than 90 mg/kg were related to samples nearby areas with oil drilling mud pits, oil production and with oil-desalting units. These areas also included samples with Pb concentrations higher than 298 mg/kg.

Dot and interpolated maps and *clr*-transformed Pb values showed the spatial distribution as modified (Fig. 9b,c). Totally, *clr*-coefficients of Pb are higher than the geometric mean of other studied metals. As higher *clr*-coefficients located from high-traffic areas and Persian Gulf Ports transit road and Ahvaz as the core of oil field activities near to Abadan and Mahshahr with large associated industries (0.78 to 1.11 or 39.4–41.79 mg/kg). It is noteworthy that agricultural fields with lower *clr* coefficients (0.54 to 0.78 or 36.68 to 39.3 mg/kg) were similar to areas with rock outcrops of sandstone and marl (− 2.4 to 0.6) or (36.68 to 39.03 mg/kg). The remaining area in the Khuzestan plain had lower to medium value for *clr*-coefficients (− 1.58 to − 0.32 or 10.4 to 31 mg/kg).

The dot map of Cu (Fig. 10a) showed that around 10 percent of samples have a high concentration (> 200 mg/kg) and corresponded to the urban area of Khuzestan plain and agricultural lands. The most elevated area (> 417 mg/kg) was observed in Ahvaz, Mahshahr, Abadan, resulting from oil desalting and production units, as well as active oil well drilling. *Clr*-transformed data dot map of Cu (Fig. 10b) had a similar distribution of raw data, while transformed data (*clr*-value) especially the highest concentration observed in the area such as Ahvaz, Mahshahr, Abadan and similar oil related areas.

Dot and interpolated maps of Cu (Fig. 10a,b), clearly showed that the urban area of Ahvaz, Abadan, Mahshar and some areas of the studied area had concentrations higher than 190 to 450 mg/kg. concentrations between 451 and 860 mg/kg restricted to areas with oil well drilling activity in oilfields, petrochemicals, steel industries, and refineries.

The highest continuous concentration of Cu (0.15–0.31 or 30.7–34.9 mg/kg) was observed in the northwest area, denoted as the dot in *clr*-transformed maps. The highest *clr*-coefficient (> 0.98 or > 34.9 mg/kg) could be observed as the dot in the middle of the studied area. In contrast, the eastern part, (nearby area with rocky outcrops and sand) had a lower value than *clr* coefficient (− 0.1 < or < 16 mg/kg) which is lower than two other metals.

As observed in the dot map of Zn (Fig. 11a), about 10 percent of samples had a high concentration (> 67 mg/kg) which are associated with the urban areas, petrochemical, and petroleum industries expanded toward the plains and agricultural lands. The interpolated map (Fig. 11) illustrates the high Zn areas with the restricted pot. Soil Zn concentrations were greater than 819 mg/kg in industrial cities such as Ahvaz, oil, and gas drilling areas like Omidieh, Mahshar, Sarbandar, and Abadan.

The dot map of *clr*-transformed Zn showed that this metal had the highest concentration between studied metals, confirming the positive frequency values of zinc *clr*-coefficient Fig. 11. The highest value of the Zn *clr*-coefficient (0.6–1 or 62.1–73.2 mg/kg) essentially occurs in the carbonate rock mass. Areas such as agricultural lands and newly developed urban areas around Ahvaz and places covered by windblown sand had the minimum *clr*-coefficient (0.3–0.9 or 20.7–54.2 mg/kg), which were lower than the geometric mean of studied PTEs.

## Discussion

Based on results in previous studies (Cicchella et al., 2005; Nazarpour et al., 2016), the high Pb concentration in raw data for urban areas, especially in the ‘old town’ was due to the use of Pb-based fossil fuels until recent years in Iran^77^. Lead is persistent in soil and its high concentrations could remain in the soil indefinitely^78^. The high amount of Pb *clr* concentrations between the northern and western parts of the studied area may be related to the combination of geogenic and anthropogenic effects (e.g. carbonate and gypsum rocks, traffic and industry and oil well drilling activities, petrochemical industry and steam power plants). The high constant of Pb *clr*-coefficient around Ahvaz and to wider urban areas might be due to the frequency of oil, steel and pipe industries which is different from area that covered by sand which have a low *clr*-coefficient -0.6–2.4 or 3.1 to 42.7 mg/kg. A similar area with low amounts of Pb according to interpolated maps used raw data and *clr*-transformed to determine in the eastern reach of the study area and some areas where rock and sandstone outcrops exist and far from oil industrial activities.

The higher amount of Cu concentration (> 190 mg/kg) in central parts of Khuzestan, corresponded to the presence of agriculture lands and weathering gypsum formations in the upstream region^79–81^. In the central to southern part of Khuzestan, a high concentration of Cu (> 190 mg/kg) was scattered and observed in areas with extensive agricultural (mostly sugarcane industry) and industrial lands. Median and high concentrations of Cu (111 to 860 mg/kg) might be related to anthropogenic sources in the metropolitan area of Ahvaz, Abadan, and Mahshahr. The highest concentration of transformed Cu *clr* (> 0.15 and 35 mg/kg) is around petrochemical and oil refinery industries, oil desalination units, and observed in small parts of the southern region.

The spatial distribution of Zn indicated a high anthropogenic influence in some limited and localised urban areas, while the spatial distribution of *clr*-coefficient was mainly affected by anthropogenic and geogenic features. Unlike two other elements, high *clr* Zn concentration (> 0.46 or 30.1 mg/kg) located in the west and north parts. In this area, Zn (50 mg/kg) was higher than Cu (15 mg/kg) and Pb (5 mg/kg), which might be due to Zn containing carbonaceous alluvium deposits. In eastern Khuzestan, despite two other metals, the *clr*-coefficient of Zn was lower than the geometry means of all three elements.

The absolute concentrations of the three studied metals exhibited an association with high anthropogenic pollution in Ahvaz, Abadan, Mahshar and some small surrounding cities, such as Susangerd. Although the anthropogenic effect is not apparent at the converted concentrations of Pb, Ahvaz, Dezful, and Susangerd showed these effects properly. Traffic intensity in this area caused Pb concentration increment rather than Cu and Zn. Finally, from the raw data concentration and spatial distribution of three studied metals, one can derive information from location of samples and area with high anthropogenic pollution. The spatial distribution of the three elements clearly show that the main pollution in the Khuzestan plain is principally concentrated in large cities, including Ahvaz, Abadan, Dezful, Mahshahr, Sarbandar and Omideh.

Evaluation geochemical factors showed that *clr*-coefficient maps might corresponded to the main geological structures: (1) high Pb values related to low Zn concentration in the western and southern parts of the study area; (2) the high amount of Pb and Zn in oil and petrochemical industries; (3) high amount of Pb in the western part especially including Susangerd and Hur-Al-Azim wetland with active drilling oil wells; (4) low amount of Pb and high Cu in agricultural areas; (5) the high amount of Cu upstream contains gypsum lithology and gypsiferous soils, and (6) low and high amount of Cu and Zn respectively in the area covered by windblown sand.

**Figure 12:**
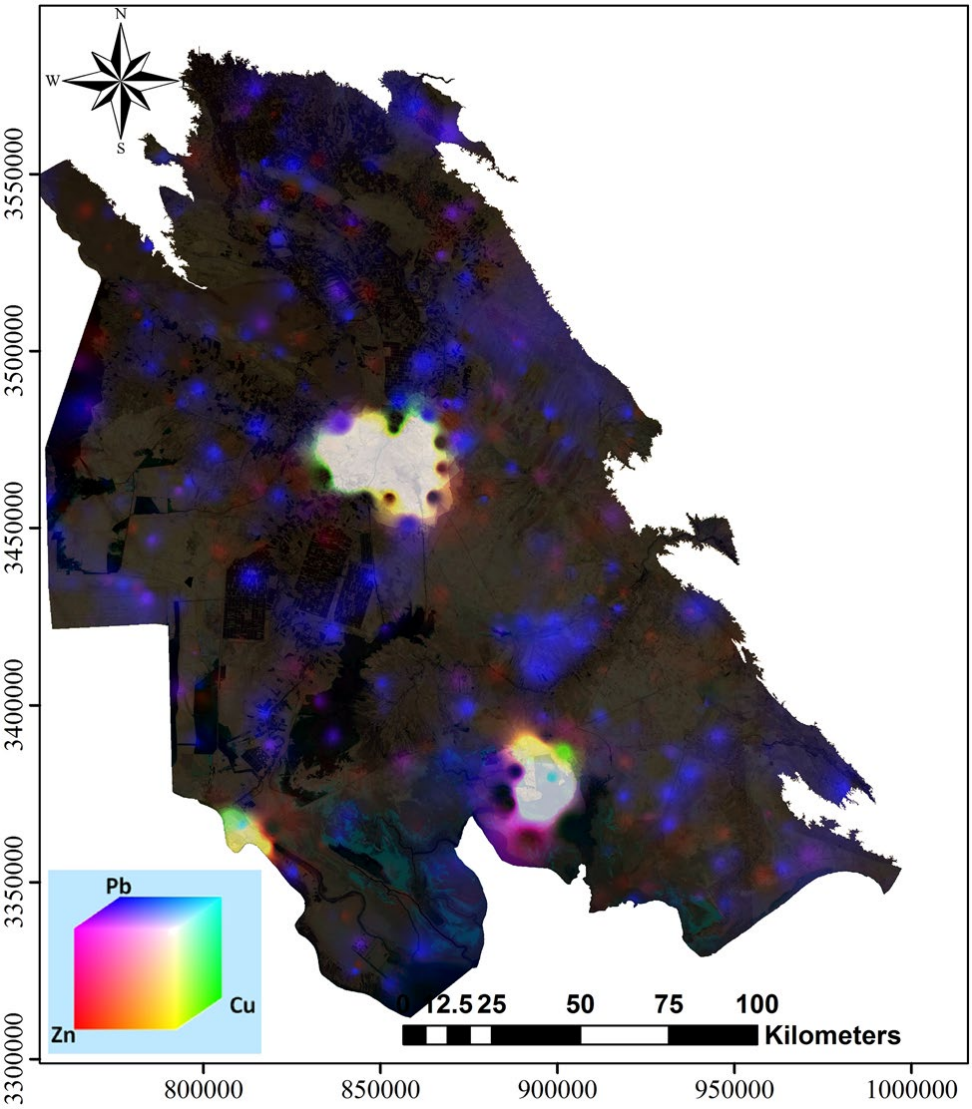
RGB composite map of Pb, Cu and Zn contamination maps. Pb, Cu and Zn distribution maps were first divided for the corresponding element concentration thresholds established by the Iranian legislation, the image was made by ArcGIS10.2.

The RGB composite map effectively distinguishes contaminated areas related to different pollution sources. Agriculture and especially farming industry activities are between the main contaminations factors to Cu in the areas with oil drilling (Fig. 12). Several studies showed Pb, Zn and Cu enrichment in surface soils near to agricultural production shops^82,83^. A lack of waste and activity management may have led to Zn and Cu contamination, shown in yellow for this area. The origin of Cu in agricultural lands is probably geogenic and resulted from intensive hydrothermal activities in the area^68,84^. The main source of Pb, Cu and Zn contamination (Purple) in Ahvaz and Mahshahr is likely due to intense car traffic and the existence of oil, petrochemical and refinery industries in these areas. High Pb concentration, as revealed by^85^ in the southern plain of the study area corresponded to anthropogenic sources related to the illegal burning of municipal waste. Ahvaz city is represented as a white colour, indicating high concomitant concentrations of all three studied metals in the region as a result of industrial and traffic pollution.

## Conclusion

In this study, statistical patterns and spatial distribution of Pb, Zn and Cu were evaluated in surface soil of the Khuzestan plain. The analytical comparison was performed between the manipulated distribution pattern of raw data and clr-transformed data. Both approaches were valuable due to revealing the true structure of the multivariate data and a new perspective on data analytical results. Raw and clr-transformed data showed different spatial distribution. Raw data distribution mainly indicates points with anthropogenic pollution, while clr-transformed data distribution could determine anthropogenic and geogenic causes effectively. This study clearly indicates that spatial distribution of raw and *clr*-transformed data should be carried out when studying spatial distributions analysis of PTEs, as different processes could be highlighted by analysing separately raw and *clr*-transformed data.

The Clr-coefficient of Pb in the studied area was associated with terrestrial and anthropogenic effects. The high concentration of Pb was observed in Ahvaz, Mahshahr and Abadan urban area as well as active zone in oil well drilling and operating activities. In addition, the *clr* value of transformed data, and geometric mean of Pb concentration was greater than Cu and Zn. Geometric studies could be determined by *clr* maps. The Pb amount is relatively low in areas covered by sandstones and windblown sand. The high amount of raw and *clr*-transformed data for Cu corresponded to farming activities, especially sugarcane in this region. Farming industry activities were highlighted by the high value of *clr*-transformed data. The high value of *clr* data in the central and south part of the studied area is mainly related to sugarcane cultivation and cultivated areas.

*The* *Clr*-coefficient map of Zn indicated a high amount of this metal in carbonate sediments for the area and anthropogenic activities in the urban area of Ahvaz, Abadan, and Mahshahr make it difficult to discriminate the low pollution inputs. This study showed to investigate the pattern of element distribution, the spatial distribution of raw and *clr*-transformed data should be analysed separately with different processes on raw and *clr*-transformed data. In addition, an RGB composite map is helpful in order to distinguish polluted areas in comparison to baseline concentrations and alongside the standard soil threshold of Iran to assess and mitigate anthropogenic and terrestrial pollution sources.
